# A Case of Chylothorax in Non-Hodgkin Lymphoma

**DOI:** 10.7759/cureus.71957

**Published:** 2024-10-20

**Authors:** Darshini V, Abdul Majeed Arshad, Irfan Ismail Ayub, Thangaswamy Dhanasekar

**Affiliations:** 1 Pulmonology and Critical Care, Sri Ramachandra Institute of Higher Education and Research, Chennai, IND

**Keywords:** chylothorax, lactate dehydrogenase (ldh), non-hodgkins lymphoma, pleural effusion, pleural fluid (pf)

## Abstract

Non-traumatic chylothorax is the abnormal collection of chyle in the pleural space without associated trauma to the thoracic duct. Untreated chylothorax is linked to serious complications and high mortality. A 68-year-old male with a five-year history of systemic hypertension presented with a two-month history of cough, dyspnea, and weight loss. He received seven cycles of chemotherapy indicated for non-Hodgkin lymphoma (NHL) of the left cervical lymph node diagnosed in 2011. Clinical examination revealed generalized lymphadenopathy, a stony dull note on percussion, absent breath sounds on auscultation over the right hemithorax, and splenomegaly on abdominal examination. Blood investigations were normal. Chest radiography showed the right pleural effusion with no mediastinal shift. Positron emission tomography scan revealed right pleural effusion with pleural thickening, mild ascites, mediastinal, axillary, and abdominal lymphadenopathy. Right-sided thoracocentesis revealed a milky white liquid, and analysis showed exudative, lymphocytic, low adenosine deaminase with high triglyceride and no malignant cells. A biopsy of a right inguinal lymph node confirmed an NHL. He was placed on second-line chemotherapy along with dietary fat restriction to medium-chain fatty acids. However, he died in December 2022. Chylothorax in NHL has extensive differential diagnoses, and diagnosis is most often delayed. An interdisciplinary treatment approach will save time and reduce mortality in such conditions.

## Introduction

Chylothorax is the accumulation of chyle, a lymphatic fluid, within the pleural cavity, which is the anatomical space between the lungs and the chest wall [1]. This fluid generally has a high concentration of fat and lymphocytes. This disorder is uncommon and could occur secondary to trauma, including chest trauma or surgeries (thoracic surgery), or during diseases like lymphoma or other tumors that block the thoracic duct [2]. In the past, non-traumatic chylothorax was frequent, but recently, traumatic chylothorax has become more common, making up for over 50% of the cases reported [3]. Less common non-traumatic etiologies include other malignancies (eg, lung cancer, Kaposi sarcoma, myeloma), infections (eg, tuberculosis, filariasis), superior vena cava (SVC) thrombosis, lymphangioleiomyomatosis, sarcoidosis, and fibrosing mediastinitis [4]. In India, the exact incidence of chylothorax is not well documented; moreover, chylothorax presents as a significant clinical challenge with several factors contributing to its complexity [5].

Chylothorax is associated with lymphomas, particularly non-Hodgkin lymphoma (NHL). NHL can cause chylothorax through obstruction or compression of the thoracic duct, a major lymphatic vessel that drains chyle from the body into the bloodstream [6]. Tumors or enlarged lymph nodes can block this duct, leading to leakage of chyle into the pleural cavity. Patients with NHL-related chylothorax often present with symptoms such as difficulty breathing (dyspnea), chest pain, and cough [7]. The accumulation of chyle in the pleural space exerts pressure on the lungs and limits their ability to expand. A biopsy of an affected lymph node can confirm the diagnosis of NHL [8]. However, there is often a delay in diagnosis due to a lack of awareness and the nonspecific nature of symptoms, which mimics other pleural effusions [9].

Limited access to advanced imaging techniques and specialized laboratory tests in rural and under-resourced areas can hinder timely and accurate diagnosis [10]. There is a need for increased awareness and training among healthcare professionals to recognize and effectively manage chylothorax. More data collection and research are essential to understand the true burden of chylothorax in India and develop targeted public health strategies.

Understanding the association between chylothorax and lymphoma is crucial for timely diagnosis and appropriate management, ultimately improving patient outcomes. Therefore, the present case report will help strengthen the existing knowledge of chylothorax and NHL.

## Case presentation

A 68-year-old man presented with a two-month history of cough, dyspnea, and weight loss and had a history of hypertension for five years. He denied fever, hemoptysis, or other constitutional symptoms. In 2011, immunohistochemistry of a biopsied left cervical lymph node revealed elevated levels of cluster of differentiation (CD)20, CD3, and B-cell lymphoma (Bcl)-2. He was then diagnosed with NHL and received seven cycles of chemotherapy, which included rituximab, cyclophosphamide, vincristine sulphate, and prednisone. On clinical examination, there was generalized lymphadenopathy, a stony dull note on percussion and absent breath sounds on auscultation over the right hemithorax, and mild splenomegaly on abdominal examination. Table 1 shows the laboratory values of pleural fluid. 

**Table 1:** Laboratory values of pleural fluid

Pleural fluid parameters	Patient Value	Reference Range
White Blood Cell Count	1105 cells/cu.mm	500-1000 cells/cu.mm
Polymorphonuclear cell count	112 cells/cu.mm	-
Polymorphonuclear cell percentage	10 %	-
Mononuclear cell count	993 cells/cu.mm	-
Mononuclear cell percentage	90 %	-
Adenosine deaminase	28.3	<33
Lactate dehydrogenase	329 U/L	<40 U/L
Triglyceride	745 mg/dl	< 150 mg/dl
Cholesterol	117 mg/dl	< 200 mg/dl
Sugar	94.5	70-110
Protein	4.2 g/dl	Less than 1.5 g/dL

**Figure 1:**
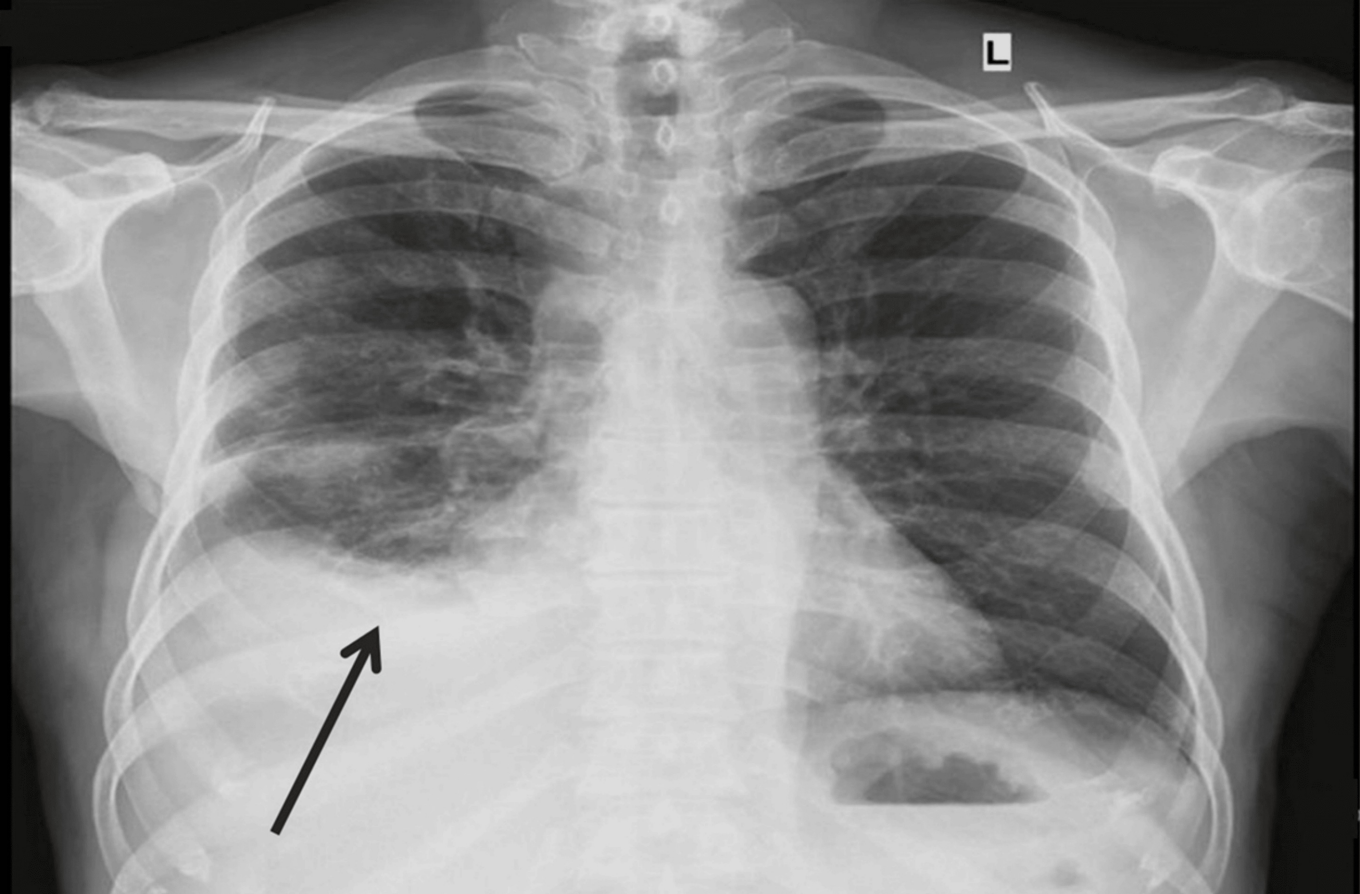
Chest radiograph showing right pleural effusion with no mediastinal shift

As shown in Figure 1, a chest X-ray performed at admission showed a right pleural effusion with no mediastinal shift. The patient's pleural fluid was drained, and it looked cloudy and milky white. Serum triglyceride and cholesterol levels were 745 mg/dL and 117 mg/dL, respectively.

**Figure 2:**
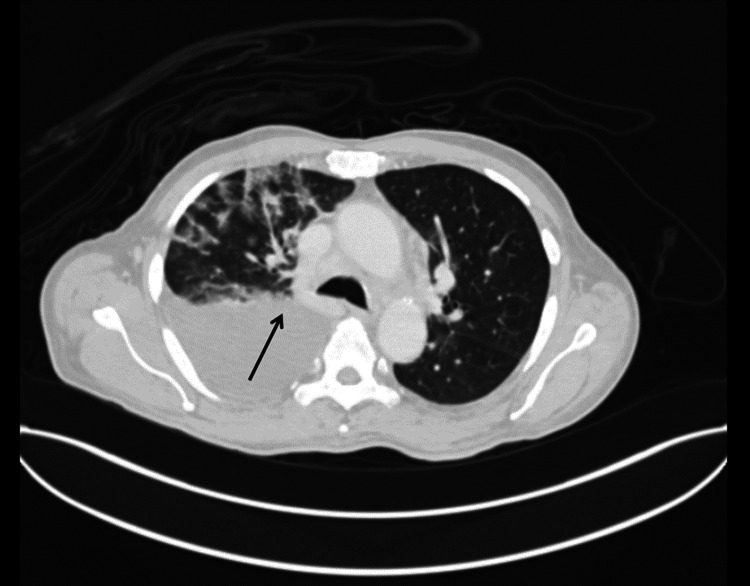
PET CT showing right pleural effusion with pleural thickening

Further cell-based studies revealed lymphocytosis but no cancerous cells. Further imaging with PET-CT revealed right pleural effusion with pleural thickening, mild ascites, and mediastinal, axillary, and abdominal lymphadenopathy as seen in Figure 2. The SUV max value above 4 was noted in the right and left paratracheal nodes, subcarinal region, bilateral scapulae, bilateral ribs, and right distal clavicle regions. The PET-CT findings indicate that the Deauville score is 4, showing increased metabolic activity.

A biopsy of the right inguinal lymph node was performed, which revealed a mixed lymphoid cell composition. The lymphoid cells had a significant proportion of CD 20-positive B-cells, while the proportions of CD 3 and BCl-2 were rather small. To initially decrease chyle production, the patient was started on second-line chemotherapy in addition to a diet that limits the intake of medium-chain triglycerides. However, the pleural effusion and respiratory problems relapsed. Upon multidisciplinary discussion, the clinicians stopped adriamycin which he was receiving in previous cycles and opted for chemotherapy combination drugs including rituximab, cyclophosphamide, vincristine sulfate, and prednisone (R-CVP). However, the patient’s quality of life deteriorated and he died in December 2022.

## Discussion

The clinical aspects of a chylothorax are determined by the degree of lymph fluid leakage and the symptoms of the underlying cause. The signs and symptoms of chylothorax are similar to those of pleural effusion. However, malnutrition and electrolyte imbalances might occur since lymph contains protein, fat, electrolytes, and vitamins [11].

Chylothorax is often associated with lymphoma when enlarged lymph nodes obstruct the thoracic duct or collateral vessels in the body [12,13]. This leads to symptoms such as dyspnea and chest discomfort in later stages, with no fever or chest pain because lymph fluid causes no pleural inflammation [14].

The gross appearance of the fluid is neither sensitive nor specific enough to diagnose a chylothorax [11]. 4 ml of turbid pleural fluid was obtained in this case. The precise mechanism of this heterogeneity in the color of chylous effusions is unknown; however, it is conceivable that it is associated with the nutritional status of the patient or underlying disorders, which may be influenced by variable lipid ingestion.

The fluid found in chylothorax is typically white and odorless; however, certain studies suggest that the fluid can sometimes appear yellowish or cloudy with traces of blood. Chylothorax can resemble empyema, but the high lipid content of chylothorax is what gives it its milky white appearance, whereas empyema has a cloudy appearance due to the buildup of blood cells, bacteria, and cellular waste. Centrifugation can be used to differentiate between these two fluids. On pleural fluid analysis, chylothorax is often exudative; however, in some instances, it may be transudative. In the present case, the pleural fluid was exudative with raised white cell count, total protein (4.2 g/dL), triglycerides (745 mg/dL), and lactate dehydrogenase (LDH) (329 mg/dL) levels. This does not discount the possibility of chylothorax, and other causes of exudates should be assessed. The gold standard for the diagnosis of chylothorax is lipoprotein analysis to detect chylomicrons in the effusion. Usually, chylomicrons are only found in lymph and are a distinguishing feature in chylothorax [15].

Malignancy is the most prevalent cause of non-traumatic chylothorax. Non-Hodgkin lymphoma is the most common cause of malignant chylothorax, accounting for up to 75% of instances. A chylothorax is diagnosed when the pleural fluid triglyceride levels exceed 1.24 mmol/L (>110 mg/dL) and the cholesterol level is less than 5.18 mmol/L (<200 mg/dL) [11]. Elevated triglycerides and cholesterol levels were noted in the present case.

Chylothorax due to duct occlusion occurs almost always on the left. If the right thoracic lymphatics are also blocked, a right chylothorax may develop [16]. Because our patient's most recent CT scans revealed no bulky disease, it seemed improbable that distinct lymph nodes could produce such extensive damage to the thoracic duct. The lymphomatous tissue may have entered the duct, causing rigidity and damage. When the duct breaks, chyle oozes into the mediastinum, subsequently the pleural cavity, and finally the parietal pleura [5].

The key discussion topic throughout therapy was to use less-invasive methods and manage the chylothorax cautiously before considering lymph node removal. In hindsight, the patient's prognosis could have been better if treatment had been carried out more rapidly upon arrival at the hospital. There are presently no defined recommendations or globally acknowledged techniques to treat chylothorax. This is mainly due to several patient-specific clinical characteristics such as the etiology and symptoms of the chylothorax, daily chyle output, and other parameters [15]. Usually, conservative treatment is done initially before more invasive treatments are performed. A conservative strategy consists primarily of dietary adjustments and effusion drainage. However, it is vital to highlight that prolonged drainage might result in immunocompromise, malnutrition, and electrolyte abnormalities [11].

## Conclusions

Chylothorax is a rare condition that can mimic empyema. The commonest non-traumatic cause of chylothorax is lymphoma. NHL has a higher incidence of chylothorax compared to Hodgkin lymphoma. Often, an extensive differential diagnosis is required, and accurate diagnosis is typically achieved only after numerous investigations. Hematologists, radiation oncologists, and thoracic surgeons should work together promptly to treat lymphoma-associated chylothorax. This will save time and lower the risk of death from this condition. More research needs to be conducted on the relationships between chylothorax and non-Hodgkin lymphoma. This should be done through multicentric studies with larger sample sizes.
